# Design and evaluation of a siloxane–amine hybrid additive for high-performance eco-friendly UV-curable anticorrosive coatings on mild steel

**DOI:** 10.1039/d6ra05788h

**Published:** 2026-09-17

**Authors:** Ehab S. Gad, Abeer A. El-Segaey, Mohamed A. Abbas, M. Attia, Sulaiman Aloraini, Mohamed A. Shenashen

**Affiliations:** a Department of Chemistry, College of Science, Jouf University Sakaka 72341 Aljouf Saudi Arabia esgad@ju.edu.sa; b Petroleum Applications Department, Egyptian Petroleum Research Institute (EPRI) Nasr City Cairo Egypt mohamedabbas1966@yahoo.com; c Research and Development Lab, Jazeera Paints Company P. O. Box: 10th of Ramadan City-Area A5 Egypt; d Chemistry Department, Faculty of Science (Men's Campus), Al-Azhar University P. B. 11884 Nasr City Cairo Egypt; e Department of Chemistry, University of Manchester Oxford Road Manchester M13 9PL UK; f Department of Chemistry, College of Science, Qassim University Buraidah 51452 Saudi Arabia; g Petrochemicals Department, Egyptian Petroleum Research Institute (EPRI) Nasr City Cairo Egypt mashenashen@gmail.com

## Abstract

Mild steel is widely used in industrial applications but remains highly susceptible to corrosion, leading to significant economic losses. Conventional chemical coatings and inorganic inhibitors are effective but environmentally hazardous, motivating the search for bio-based alternatives. In this study, UV-curable coatings were developed using epoxidized soybean oil acrylate (ESOA) and tripropylene glycol diacrylate (TPGDA), modified with varying concentrations of poly(di-triethanolaminesiloxane-oleate) (PDTSO). The optimized formulation (4% PDTSO) exhibited a remarkable increase in charge transfer resistance (*R*_ct_) from 83 Ω cm^2^ for bare steel to 21 000 Ω cm^2^, demonstrating a substantial improvement in corrosion resistance. Water contact angle improved from 69° to 85°, indicating reduced surface wettability. Salt spray testing further demonstrated a performance upgrade from grade 2G to 7G. These coatings also showed balanced mechanical properties, combining hardness with flexibility, due to increased crosslinking density. The results highlight PDTSO's effectiveness as a sustainable corrosion inhibitor and its potential in next-generation eco-friendly protective coatings for mild steel.

## Abstract 2

Designing bio-based UV-curable coatings as eco-friendly protective coatings for mild steel used in industrial applications.

## Graphical abstract

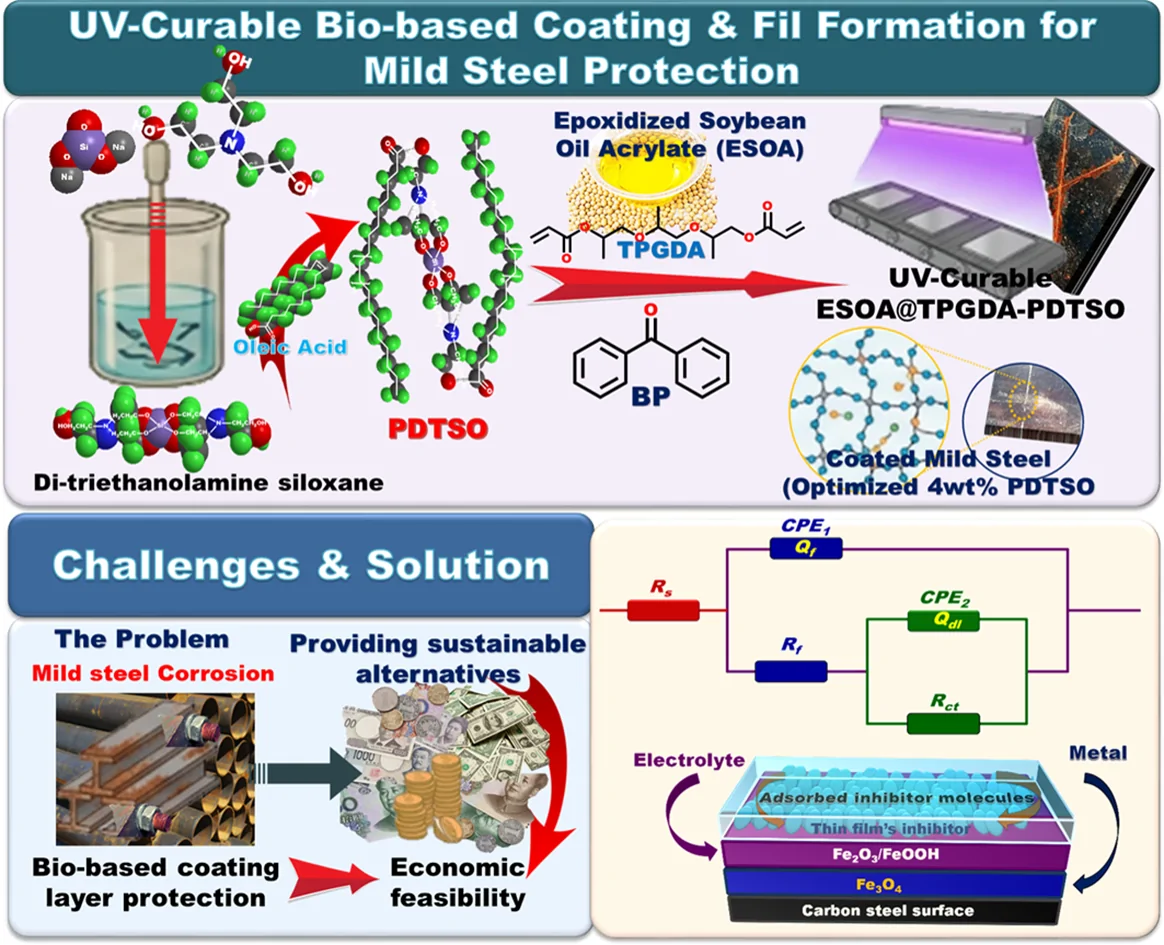

## 1. Introduction

Mild steel is the most common low-carbon steel produced worldwide and is essential to industry and the economy.^1,2^ It is extensively employed in oil refining, construction, concrete reinforcement, containers, and mechanical components.^2,3^ Despite its low cost and acceptable mechanical properties, its susceptibility to corrosion causes substantial economic losses. Corrosion costs approximately $2.5 trillion annually worldwide,^2–5^ affecting infrastructure and major industries. The petroleum industry spends $1.372 billion annually on pipeline maintenance,^6^ while marine and aviation industries incur losses of $50–80 billion and $2.2 billion, respectively.^5,7,8^

Developing corrosion control methods is a major research focus.^9^ Coatings and corrosion inhibitors are among the most effective approaches. While inorganic inhibitors such as chromates and phosphates exhibit high efficiency, their environmental risks have encouraged the development of safer alternatives.^10–12^ Organic coatings have emerged as promising candidates, particularly UV-curable systems due to their rapid curing, energy efficiency, and low environmental impact.^13–17^

The environmental concerns associated with petrochemical-based coatings have increased interest in renewable resources.^18^ Biomass-derived materials are attractive because of their abundance, biodegradability, low cost, and strong adhesion to metal surfaces.^19–22^ Vegetable oils contain reactive functional groups suitable for polymer synthesis.^23,24^ Among them, castor oil is particularly attractive because its hydroxyl-rich structure enables the preparation of durable and chemically resistant polymers.^25^

Their durability, surface energy, and thermal stability make silicone materials valuable.^26,27^ Consequently, organosilicon polymers are widely used in anti-corrosion coatings. UV-curable silicones combine the advantages of UV curing with the functional properties of silicones, making them suitable for coatings, self-cleaning surfaces, and other advanced applications.^26,27^ Bio-based UV-curable siloxanes further provide environmentally friendly, energy-efficient coatings with reduced VOC emissions and enhanced anti-corrosion performance.^28–32^

By functionalizing SiO_2_ with octadecyl amine using γ-glycidylethoxypropyltrimethoxysiane, Song *et al.*^33^ developed a hydrophobic ODA–SiO_2_/KSA coating using a silicone-acrylic emulsion (KSA) prepared *via* MPS-functionalized polymerization, with improved durability and corrosion resistance. Similarly, epoxy–polysiloxane hybrid nanomicelles exhibited enhanced adhesion, mechanical strength, and barrier properties, making them suitable for marine applications.^34–37^

Despite progress, UV-curable systems still suffer from oxygen inhibition during free-radical polymerization. Tertiary amines and oxygen scavengers have been employed to overcome this limitation, with 2,4,6-tris(dimethylaminomethyl)phenol showing excellent room-temperature co-curing performance.^34,38^ Soybean oil-derived epoxidized soybean oil acrylate (ESOA) has also attracted considerable attention for UV-curable coatings because of its renewable origin.^38–40^ Although high-performance ESOA systems have been reported, their relatively high viscosity and limited mechanical properties continue to restrict broader applications.^28,38,41,42^

This work explores the incorporation of varying proportions of PDTSO into ESOA@TPGDA-based UV-curable coatings as a novel corrosion-inhibiting strategy. Unlike conventional approaches, PDTSO is employed not only as a functional additive but also as an active contributor to network formation and surface modification. The study focuses on elucidating the role of PDTSO in tailoring crosslinking behavior, surface characteristics, and overall coating performance within a sustainable polymeric framework. By integrating renewable ESOA with PDTSO-modified networks, this work aims to provide a deeper understanding of structure–property relationships in advanced protective coatings, offering a promising pathway toward environmentally friendly and long-term corrosion protection systems.

## 2. Experimental

### 2.1. Materials

All chemicals were used as received, without further purification. Sodium metasilicate (Na_2_SiO_3_, ≥95%), triethanolamine (TEA, ≥98%), oleic acid (≥98%), *p*-toluenesulfonic acid (pTSA, ≥97%), and benzoyl peroxide (BPO, ≥98%) were purchased from Sigma-Aldrich. Absolute ethanol (≥99.8%) and diethyl ether (≥99.5%) were supplied by Adwic (Egypt).

The UV-curable matrix consisted of (i) epoxidized soybean oil acrylate oligomer (ESOA, EBECRYL® 5848), (ii) tripropylene glycol diacrylate (TPGDA, reactive diluent), both supplied by Allnex (Belgium); and (iii) benzophenone (BP, ≥99%), used as a photoinitiator, obtained from Ciba Specialty Chemicals (Switzerland). Mild steel coupons (ASTM A36 equivalent) were provided by the Iron and Steel Company (ISC), Egypt. Their nominal composition (wt%): C 0.19, Mn 0.94, Si 0.05, *P* ≤ 0.01, *S* ≤ 0.005, with residual Cu, Ni, Cr, V, and Ti (<0.03% each), balance Fe.

### 2.2. Synthesis of di-triethanolaminesiloxane (DTS)

To synthesize DTS, a condensation reaction was performed in a three-neck flask under nitrogen. Triethanolamine (14.9 g, 0.1 mol) and anhydrous sodium metasilicate (6.0 g, 0.049 mol) were suspended in ethanol and heated at 120 °C for 2 h, followed by 250 °C for 2 h.^43^ The reaction proceeds *via* silanol condensation and nucleophilic substitution at silicon centers: hydrolysis of metasilicate generates Si–OH groups, which either self-condense or undergo nucleophilic attack by TEA hydroxyls, with water as the sole by-product. After cooling, the product was redissolved in ethanol, precipitated into cold diethyl ether, filtered, washed, and dried under vacuum, affording DTS in 82% isolated yield.

### 2.3. Synthesis of poly(di-triethanolaminesiloxane-oleate) (PDTSO)

PDTSO was synthesized *via* a one-pot esterification–radical polymerization sequence. DTS (6.4 g, 20 mmol) and oleic acid (11.2 g, 40 mmol) were dissolved in toluene (50 mL) and refluxed under N_2_ at 120 °C for 6 h with *p*-toluenesulfonic acid (*p*-TSA, 1 wt%) to promote esterification between residual hydroxyl groups of DTS and the carboxylic acid of oleic acid, releasing water as the sole by-product.

Benzoyl peroxide (BPO, 1 wt%) was then added, and the mixture was stirred at 120 °C for another 6 h to initiate radical cross-linking *via* addition across the C=C bonds of oleate chains.

The crude product was cooled, dissolved in ethanol, and purified by triple precipitation into diethyl ether. After vacuum drying (50 °C, 24 h), PDTSO was obtained as a viscous brown polymer in 81% isolated yield. Fig. 1 shows a schematic illustration of the PDTSO synthesis steps.

**Fig. 1:**
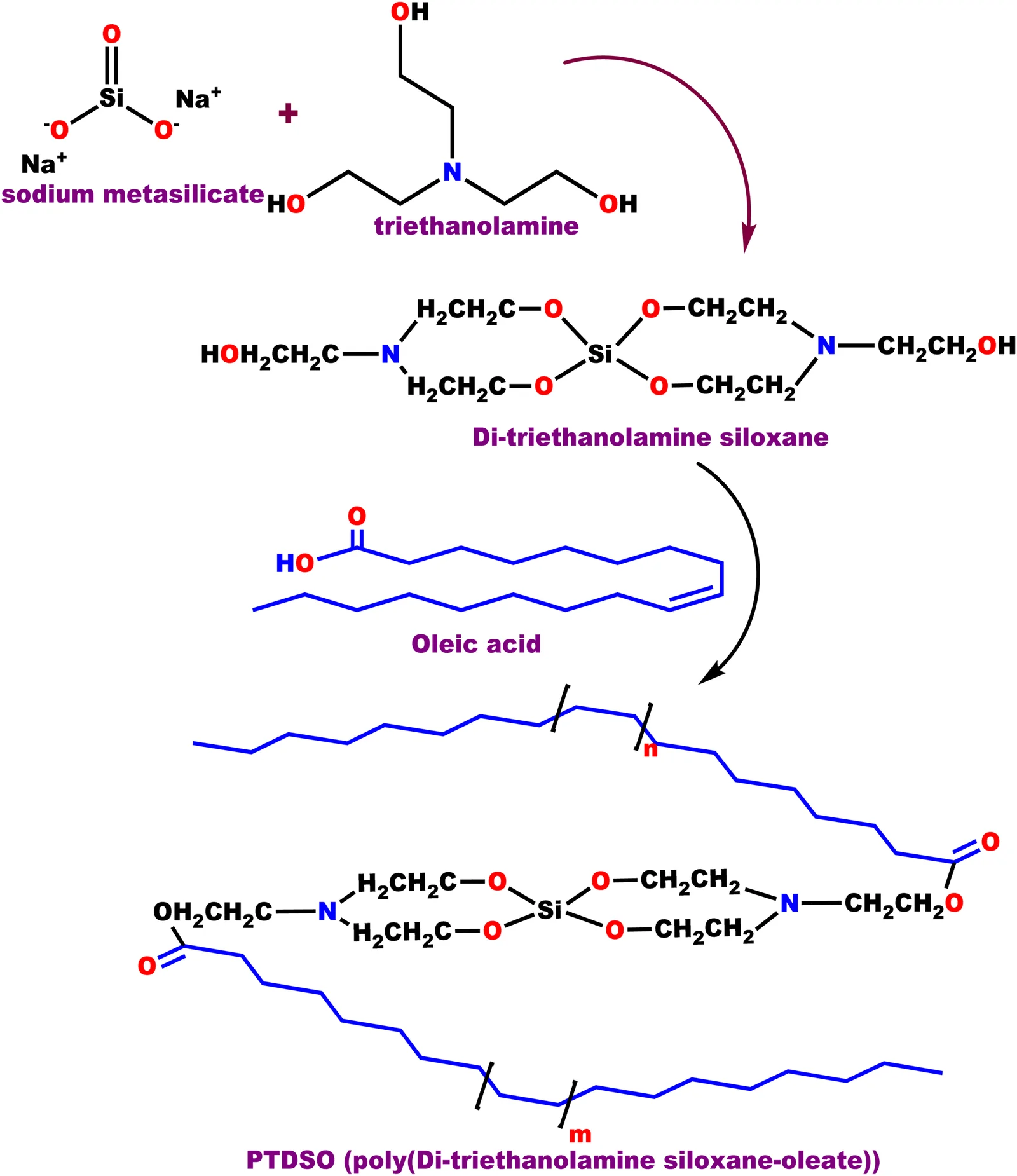
A schematic design highlighting the different PDTSO synthesis steps.

### 2.4. Preparation of UV-curable ESOA@TPGDA–PDTSO coating polymers

UV-curable coating polymers, designated as ESOA@TPGDA–PDTSO, were manufactured using a well-defined base mixture comprised of 71 wt% Epoxidized Soybean Oil Acrylate (ESOA, a bio-based oligomer known for its excellent film-forming properties, 25 wt% Tripropylene Glycol Diacrylate (TPGDA), and 4 wt% Benzophenone (BP) serving as the photoinitiator to initiate free-radical polymerization upon UV exposure. A control sample, ESOA@TPGDA, was also prepared without the inclusion of the synthesized inhibitor. To investigate the PDTSO additive impact on coating performance, various mass percentages (0, 1, 2, 3, and 4 wt%) of the synthesized PDTSO were incorporated into the control ESOA@TPGDA matrix composite. Each mixture was stirred continuously for 10 min to confirm uniform dispersion. The synthesized coatings were uniformly applied onto mild steel substrates (102 × 152 mm)) that had been mechanically polished (SiC papers: 180 → 600 → 1200 grit), ultrasonically degreased in acetone for 10 min, rinsed with ethanol, and dried under N_2_. Coating was performed using a standardized film applicator (Elcometer 3540, 100 µm gap), followed immediately by UV curing (*T*_max_, China; 80–120 W cm^−2^, 340–360 nm) for 20 s.

### 2.5. Characterization methods

The chemical composition of synthesized ESOA@TPGDA and ESOA@TPGDA–PDTSO formulations containing 0–4 wt% PDTSO was characterized by FTIR (Fourier transform infrared) spectroscopy using a Thermo-Fisher Nicolet™ iS™10 instrument (USA). Additionally, ^1^H NMR analysis was performed on a Bruker Avance 400 MHz spectrometer operating at 9.4 T. Deuterated DMSO was used as the solvent, while tetramethylsilane (TMS) served as the internal reference for chemical shift calibration and structural evaluation of the synthesized PDTSO polymer.

The crystalline structure and phase composition of the coatings were investigated by XRD (X-ray diffraction) using an X'PERT-PRO-MPD (PANalytical, Netherlands) with Cu Kα radiation (*λ* = 1.5406 Å) operating at 40 kV and 40 mA. Surface morphology was examined by SEM (scanning electron microscopy) using a Zeiss EVO 10 instrument (Germany) equipped with EDX for elemental analysis at 15 kV. Prior to imaging, samples were mounted on aluminum stubs with carbon tape and coated with a thin gold layer to improve conductivity and prevent charging.

### 2.6. Gel content and equilibrium swelling ratio analysis

The degree of crosslinking and the crosslinking density of the cured coatings were evaluated by gel content and equilibrium swelling ratio measurements, respectively. To evaluate the degree of crosslinking and the crosslinking density of the cured coatings, about 1.0 g of the cured coating sample was immersed in 20 mL of acetone at room temperature (25 ± 2 °C) for 24 hours with gentle agitation. Acetone was chosen as the extraction solvent due to its compatibility with the epoxy acrylate (ESOA) and TPGDA components, enabling efficient extraction of the uncrosslinked polymer fraction while preserving the crosslinked UV-cured ESOA@TPGDA network's integrity. Following the extraction phase, the swelled samples were separated from the solvent, the excess acetone on the surface was carefully removed using with filter paper, and the swollen weight (*W*_s_) was immediately recorded. The resulting samples were then dried for 10 hours at 50 °C in a vacuum oven until they reached a constant weight (*W*_t_), at which time the gel fraction was calculated.

The gel content (%) was calculated using the following equation:^7,9,44^
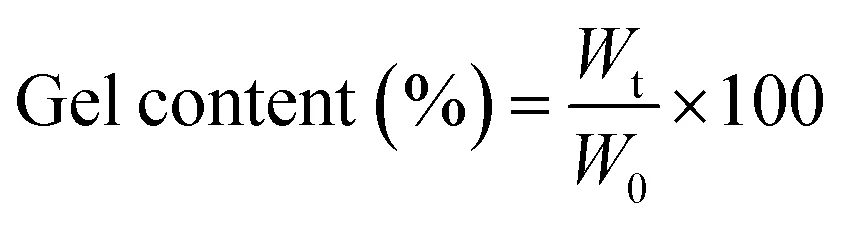
where, *W*_t_, and *W*_0_ represented the weight of dried sample after extraction, and the initial weight of the sample before immersion, respectively.

The equilibrium swelling ratio (%) was calculated using the following equation:
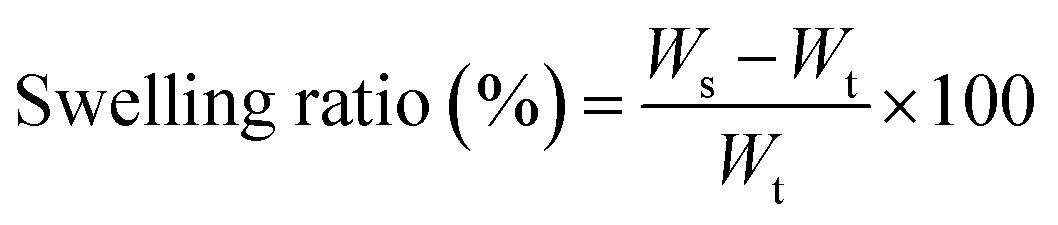
where *W*_s_ is the weight of the swollen sample after equilibrium solvent uptake and *W*_t_ is the constant dry weight after extraction.

### 2.7. Contact angle measurement

The analysis of contact angle was evaluated in order to evaluate the hydrophobicity and surface energy features of the cured coatings. The surface wettability of the UV-curable polymer coatings was evaluated using static water contact angle measurements. A Standard Goniometer, Model 21 AC, from Rame-Hart Instruments Company, New Jersey, USA was applied. Using a microsyringe, a droplet of water (about 5 µL) was carefully applied to the surface of the cured film. To reduce the effects of evaporation, the contact angle was recorded within 5 seconds.

### 2.8. Salt spray test

Corrosion resistance was assessed *via* a 500 h neutral salt spray test (5 wt% NaCl, 35 ± 1 °C) following ASTM B117 and ASTM D1654-21. Prior to exposure, each coated steel panel was scribed (25 mm, ≤1 mm wide, to substrate, 45°) using a calibrated tool. Visual inspection and photography were performed at 500 h. Final ratings (0G–10G) were assigned by two independent evaluators using ASTM D1654 reference images, with rust area quantified *via* image for validation.^45^

### 2.9. Electrochemical measurements

Electrochemical analysis is a key technique for evaluating the corrosion resistance of synthetic coatings. Polarization and impedance measurements were performed using an OrigaMaster 5 exe system integrated with Zsimpwin software. A standard three-electrode cell was employed, consisting of a UV-curable coated mild steel (MS) working electrode with an exposed area of 1 cm^2^, a platinum wire counter electrode, and a saturated calomel electrode (SCE) as the reference electrode. The electrode potential was stabilized for 1 h before measurements.^46^ Electrochemical impedance spectroscopy (EIS) was conducted using a 10 mV AC perturbation at open-circuit potential over a frequency range of 100 kHz to 50 mHz. Potentiodynamic polarization measurements were performed from −800 to −300 mV at a scan rate of 0.2 mV s^−1^.

In accordance with ASTM G3-74 and G87 standards, this range was selected to cover the linear Tafel regions for both anodic and cathodic reactions based on measured open-circuit voltage values (roughly −650 to −450 mV), while maintaining a voltage range of ±150 to 200 mV around the stable open-circuit voltage. While the −800 mV cathode limit reduces excess hydrogen emission, bubbling, current instability, and possible coating damage, the −300 mV anode limit stops passivation, pitting, and oxygen production, enabling monitoring of the active fusion zone of iron. This potential range around the corrosion potential (*E*_corr_), enabling reliable determination of the corrosion current density (*i*_corr_). By allowing sufficient time for the charges to diffuse through the coating and reach a stable state, the 0.2 mV s^−1^ scanning rate reduced the capacitive currents. Under the typical conditions of mild steel in a 3.5% sodium chloride solution, this potential range allowed for linear Tafel areas for all samples, preventing coating degradation or nonlinear polarization behavior. All electrochemical tests were carried out at 30 °C according to ASTM G3-74 and G87 standards.^10,47^

### 2.10. The physico-chemical and mechanical properties of cured coated films

Through utilizing a various standardized assessment methodology, the physico-chemical and mechanical features of the synthetic cured coating films were investigated.

➢ According to ASTM D7027 guidelines, coating hardness was evaluated by scratch resistance analysis using a Mechanized Scratch Tester (SH705, TQC Sheen, UK).

➢ The cured films' flexibility of the was assessed using a cylindrical mandrel bending test, where the coatings were bent over a 5 mm mandrel, in accordance with ASTM D522.

➢ The cured films' adhesion was evaluated *via* the cross-cut method, following the ASTM D3359 standard.

➢ Surface gloss was measured at a 60° angle using an Elcometer 480 Gloss Meter, in compliance with ASTM D523.

➢ Impact resistance was assessed using an ERICHSEN Model 304 Impact Tester, according to ASTM D2794 specifications.

### 2.11. Reproducibility of test results

All tests were performed in triplicate (*n* = 3) to assess experimental reproducibility. Results are reported as mean ± standard deviation (SD). The sample standard deviation (*s*) was calculated using:
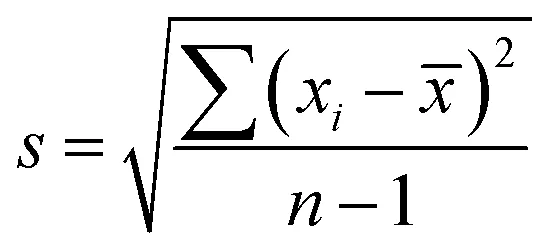
where *x*_*i*_ is the *i*-th measurement, *x̄* is the sample mean, and *n* = 3 is the number of replicates. This unbiased estimator quantifies data variability and supports the reliability and reproducibility of the coating system.

## 3. Results and discussion

### 3.1. Characterizations of the synthetic compounds

The synthetic DTS and PDTSO compounds were subjected to FTIR spectroscopy in order to examine structural changes and presence of key functional (Fig. 2A). The FTIR spectrum of DTS showed a broad absorption peak around 3400 cm^−1^ that associated with hydroxyl (–OH) groups. This peak disappeared in the PDTSO spectrum, indicating successful esterification with oleic acid. Peaks corresponding to asymmetric C–H stretching vibrations were observed between 2850 and 2900 cm^−1^, and a prominent peak at 1716 cm^−1^ confirmed the presence of carbonyl (C=O) groups. Additionally, a Si–O bond was identified at 954 cm^−1^, while a C–N stretching band appeared at 1177 cm^−1^. The peak's disappearance at 1600 cm^−1^ provided additional evidence that the polymerization and structural conversion processes were completed.

**Fig. 2:**
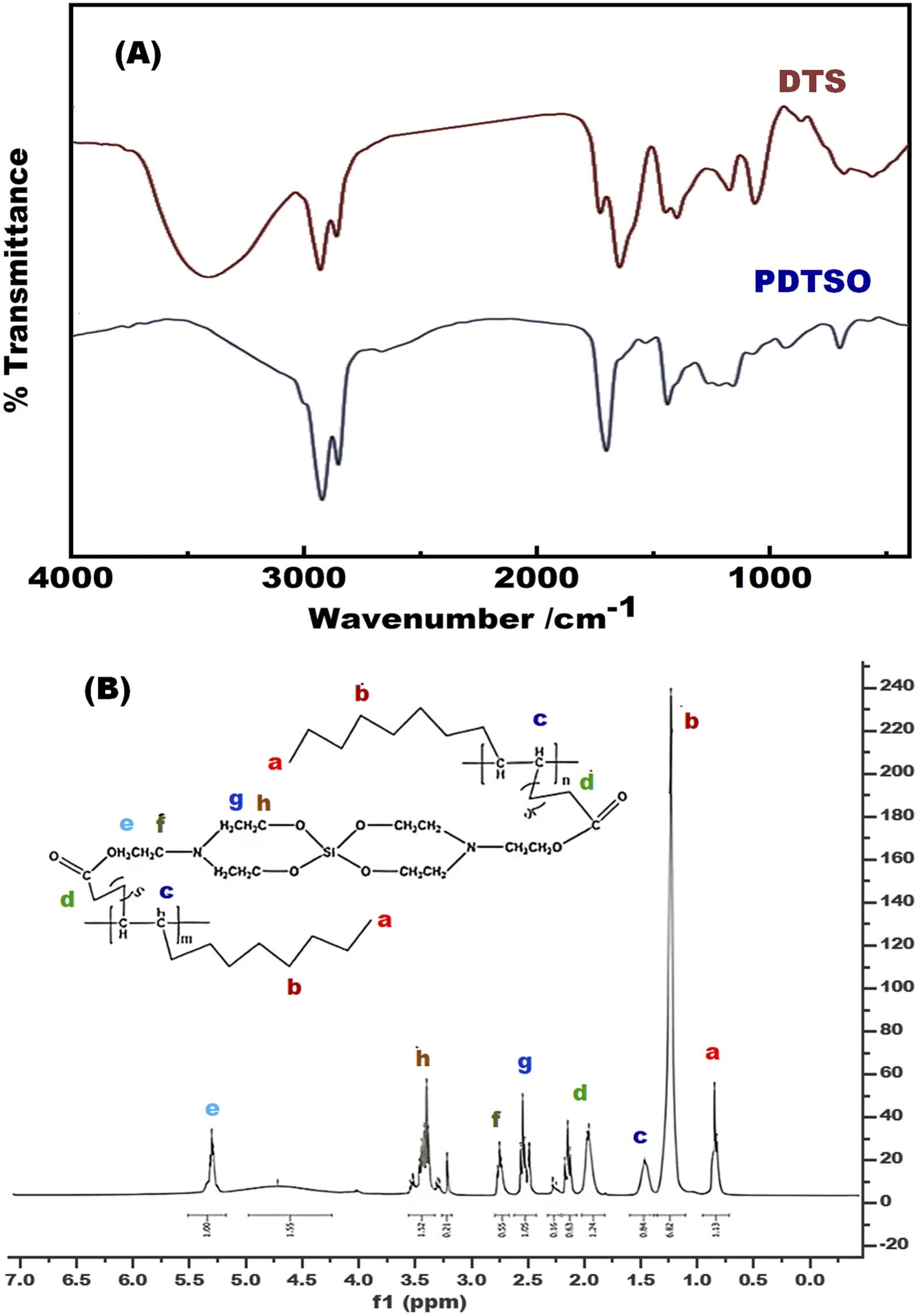
(A) Represented the FTIR spectra of the synthesis DTS and PDTSO; and (B) shows the ^1^H NMR spectrum for the synthesis of PDTSO.

^1^H NMR spectroscopy was employed to validate the structure of the synthesized PDTSO compound (Fig. 2B). The resulting spectrum exhibits characteristic chemical shifts corresponding to specific protons in the molecular structure, confirming successful polymer formation. The triplet at *δ* = 0.7–0.8 ppm (t, *J* ≈ 6–7 Hz) is associated with the terminal methyl (–CH_3_) groups of the fatty acid chain, while the multiplet at *δ* = 1.3 ppm corresponds to methylene (–CH_2_–) groups in the oleic acid hydrocarbon chain. A singlet at *δ* = 1.5 ppm is attributed to methine (CH) protons in the polymer backbone. The triplet at *δ* = 1.8–2.0 ppm (*J* ≈ 7 Hz) is assigned to methylene protons adjacent to the carbonyl group (CH_2_–CO), supporting ester formation. In addition, the multiplet at *δ* = 2.5–2.6 ppm corresponds to nitrogen-bound methylene groups (CH_2_N) derived from the DTS segment. Signals at *δ* = 3.2–3.5 ppm are assigned to oxygen-bound methylene protons (CH_2_O), confirming the presence of ether linkages. These spectral features collectively verify the successful synthesis and structural integrity of the PDTSO polymer.

Moreover, FTIR spectroscopy was employed to analyze UV-curable ESOA@TPGDA–PDTSO coating films containing different PDTSO concentrations (0–4 wt%) (Fig. 3). This technique was used to monitor structural changes and evaluate photopolymerization behavior under UV exposure.

**Fig. 3:**
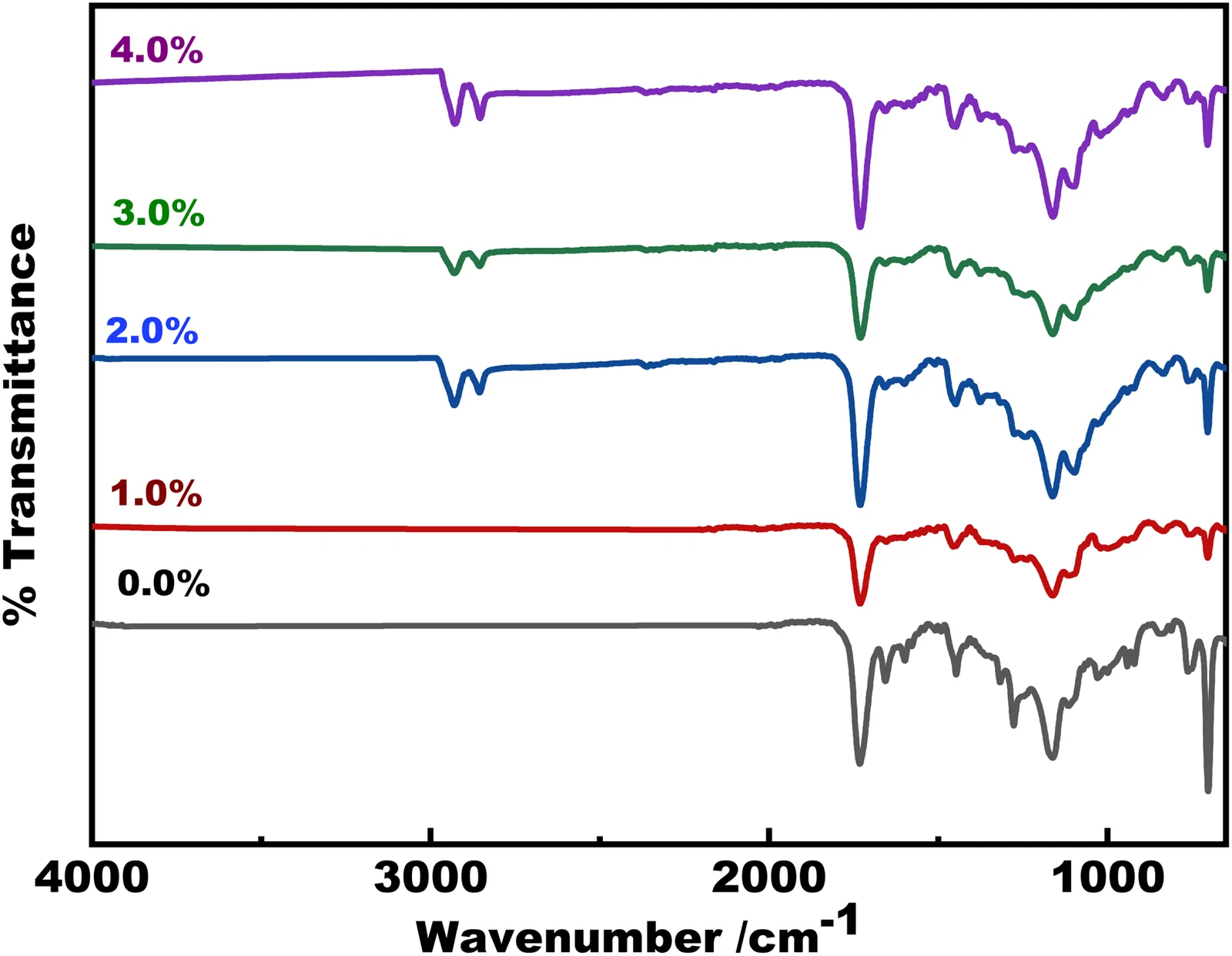
Shows the FTIR spectra of the synthesized thin films of the UV-curable ESOA@TPGDA–PDTSO fabricated at different concentrations of the PDTSO (0–4 wt%).

As shown in Fig. 3, the FTIR spectrum of the control ESOA@TPGDA matrix exhibits a strong absorption peak at 1720 cm^−1^ corresponding to carbonyl (C=O) stretching and a peak at 1174 cm^−1^ assigned to C–O stretching in triglyceride units. The peak at 1631 cm^−1^, attributed to C=C stretching, indicates the presence of residual unreacted acrylate double bonds. With increasing PDTSO content, especially at 3–4 wt%, the intensity of this peak markedly decreases, indicating greater consumption of acrylate groups during curing. This observation, together with the increased gel fraction (>93%) and improved mechanical properties, confirms the formation of a highly cross-linked network. Furthermore, the appearance of peaks around 2925 cm^−1^ in PDTSO-containing formulations is assigned to methylene (–CH_2_–) groups, confirming the incorporation of PDTSO into the polymer matrix and its contribution to network integrity.

### 3.2. XRD measurements

The short-range structural ordering and amorphous nature of UV-curable ESOA@TPGDA–PDTSO films containing 0–4 wt% PDTSO were investigated by XRD analysis (Fig. 4). The study aimed to evaluate the effect of PDTSO incorporation on molecular packing and short-range order within the cured polymer network. As shown in Fig. 4, all samples display a broad amorphous halo between 17° and 30° (2*θ*), with the diffraction maximum shifting from 19.0° to 20.3° after PDTSO addition (Table 1). This confirms the predominantly amorphous structure of the UV-cured ESOA@TPGDA matrix, with no evidence of long-range crystalline order. Therefore, the reported crystallinity values are apparent and semi-quantitative, reflecting relative changes in local molecular alignment.^48^

**Fig. 4:**
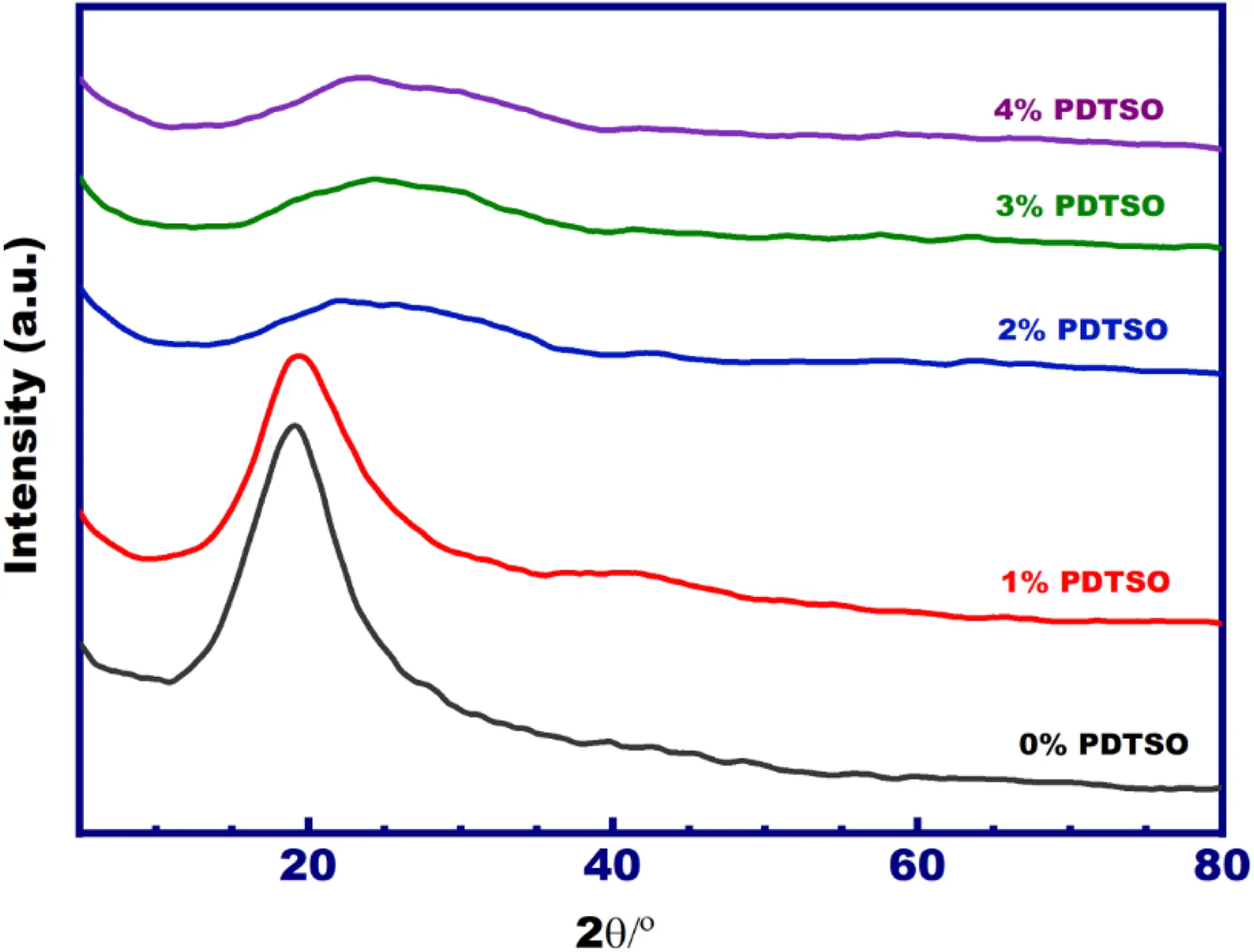
XRD patterns of the synthesized thin films of the UV-curable ESOA@TPGDA–PDTSO fabricated at different concentrations of the PDTSO (0, 1, 2, 3, and 4 wt%).

**Table 1:** Semi-quantitative XRD parameters of UV-cured ESOA@TPGDA–PDTSO coatings with varying PDTSO content

Sample	2*θ* (°)	*d*-Spacing (Å)	FWHM (°)	Apparent crystallinity (%)
0% PDTSO	19.0	4.68	6.8	18
1% PDTSO	19.3	4.62	6.4	21
2% PDTSO	20.0	4.46	6.0	24
3% PDTSO	20.3	4.40	5.8	26
4% PDTSO	20.3	4.40	5.9	25

The gradual shift of the diffraction maximum to higher 2*θ* values corresponds to a decrease in interchain *d*-spacing from 4.68 to 4.40 Å, indicating enhanced chain packing and reduced free volume due to favorable interactions between PDTSO and epoxy acrylate chains.^49^ The FWHM decreases from 6.8° to 5.8° at 3 wt% PDTSO, while apparent crystallinity increases from 18% to 26%, suggesting improved short-range molecular ordering within the amorphous phase. The slight FWHM increase at 4 wt% PDTSO (5.9°) may indicate saturation effects or minor phase separation.

Such structural refinement is expected to enhance mechanical flexibility, barrier properties, and coating integrity. Improved local ordering may reduce microstructural defects and promote more homogeneous crosslinking, contributing to better corrosion resistance and longer coating service life.^50^

### 3.3. Surface and compositional measurements

The microstructural and compositional investigation of UV-cured polymer coatings using SEM, EDS, and elemental mapping are shown in Fig. 5. Measurements of the control coating (ESOA@TPGDA, 0% PDTSO) and the PDTSO-modified coating (ESOA@TPGDA–PDTSO, 4 wt% PDTSO) are presented in Fig. 5A–F, respectively. SEM micrographs were utilized to assess surface morphology, uniformity, and PDTSO dispersion within the ESOA@TPGDA polymer matrix, providing insight into the influence of PDTSO on the microstructure of UV-curable coatings. SEM images of the control coating at different magnifications show a rough, wrinkled surface with visible defects, suggesting insufficient cross-linking, incomplete polymerization and volumetric shrinkage (Fig. 5A). This irregular texture suggests insufficient crosslinking within the polymer matrix, consistent with FTIR results. Such structural deficiencies may adversely affect adhesion, durability, and corrosion resistance, while increasing susceptibility to cracking and delamination.^51^

**Fig. 5:**
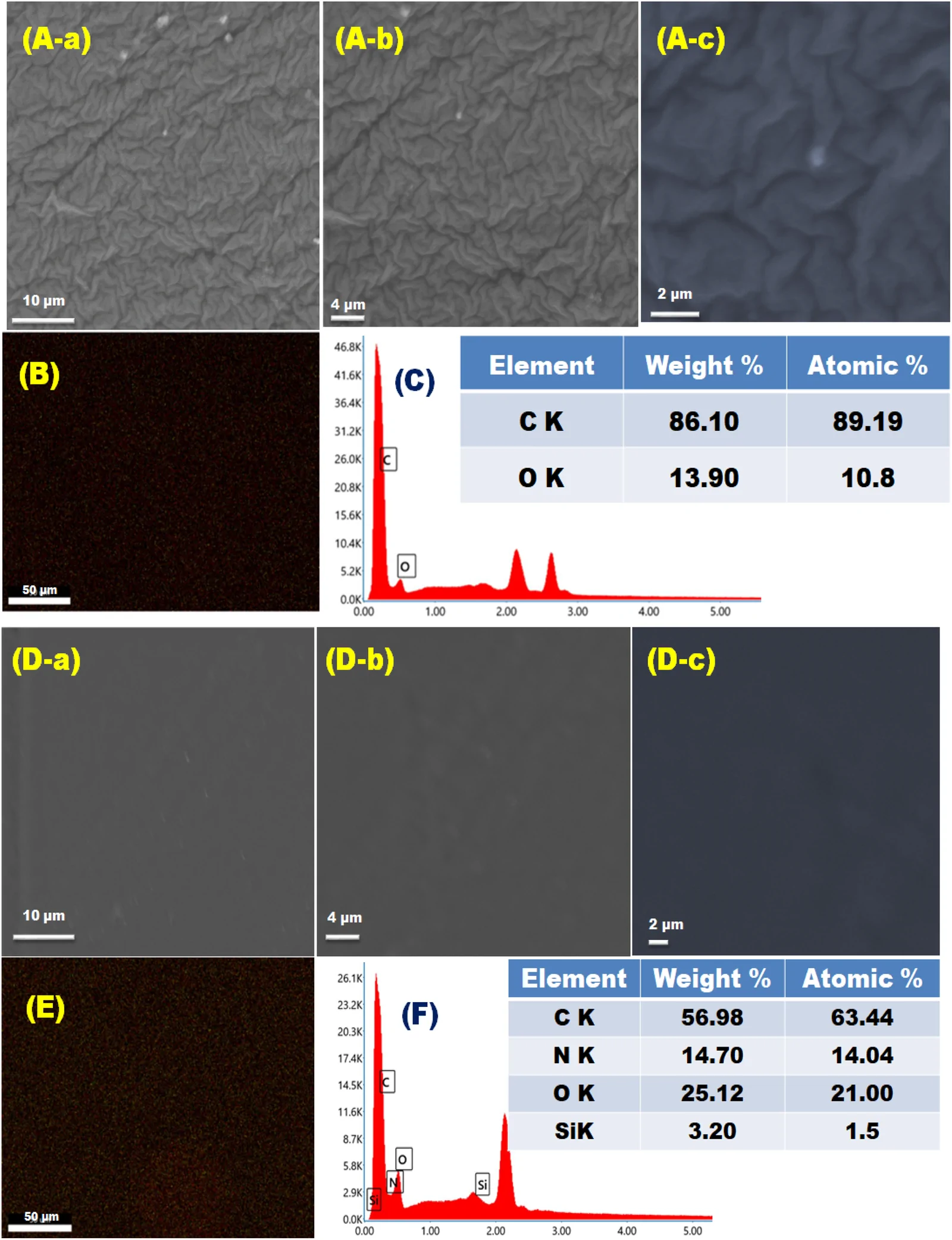
SEM imaging, elemental mapping, and EDS spectroscopy of UV-cured coatings before and after PDTSO incorporation. (A–C) Representing the control ESOA@TPGDA formulation (0% PDTSO): (A) SEM image revealing a wrinkled surface indicative of incomplete polymerization; (B) elemental mapping of C and O; (C) EDS spectrum confirming the exclusive presence of C and O. (D–F) Display the PDTSO-modified formulation (4 wt% PDTSO): (D) SEM image; (E) elemental mapping of C, O, Si, and N; and (F) EDS spectrum analysis.

In contrast, the SEM images of the PDTSO-modified coating at different magnifications exhibits a uniform, smooth surface under identical curing conditions, indicating enhanced cross-linking and improved network development (Fig. 5D).^52^ The incorporation of PDTSO reduced shrinkage-related defects and improved network cohesion, structural uniformity, flexibility, and barrier performance.

The uniform and homogeneous distribution of C and O throughout the control polymer matrix is depicted in the elemental mapping distribution image (Fig. 5B), demonstrating the coating's consistent elemental composition. Additionally, the EDS spectrum of the control coating demonstrates and validates the distinctive C and O peaks, which are compatible with and validate the predicted elemental composition of the ESOA@TPGDA polymer matrix with percentages of 86.10% and 13.90%, respectively (Fig. 5C). The elemental distribution of the PDTSO-modified coating showed a homogeneous distribution of silicon and nitrogen throughout the polymer matrix, confirming the uniform incorporation of the additive (Fig. 5E). EDX analysis showed silicon and nitrogen peaks at 1.74 kV and 0.39 kV, respectively (Fig. 5F). The results indicated the presence of C, N, O, and Si at percentages of 56.98%, 14.17%, 25.12%, and 3.20%, respectively, in the 4% PDTSO sample.

### 3.4. Gel content and equilibrium swelling ratio analysis

Gel content is a direct indicator of the degree of crosslinking in UV-cured coatings, whereas the equilibrium swelling ratio provides complementary information regarding the compactness of the crosslinked polymer network. Essentially, a higher gel content denotes a more stable and dense polymer network. Because of its stability, barrier performance is enhanced in terms of resistance to solvent effects, thermal stability, and corrosive chemical resistance. The association between gel content and corrosion resistance was verified by salt spray testing and electrochemical impedance measuring (EIS). The gel content does not accurately represent the level of structural compactness, despite being employed as a gage of network creation. In order to verify this, equilibrium swelling ratio measurements in acetone were utilized to measure entanglement density directly.

Table 2 summarizes the gel content and equilibrium swelling ratio of the ESOA@TPGDA–PDTSO coatings prepared with different PDTSO concentrations. The results revealed a systematic drop in swelling ratio of the control coating as the PDTSO content increased, indicating a denser network with less free volume. The pristine ESOA@TPGDA coating exhibited the lowest gel content (84.0 ± 0.8%) and the highest swelling ratio (28.0 ± 0.7%), indicating a lower degree of crosslinking and a relatively loose polymer network. With increasing PDTSO content, the gel content progressively increased to 95.0 ± 0.4%, while the equilibrium swelling ratio decreased to 7.0 ± 0.3%. The decrease in solvent uptake indicates the formation of a more compact crosslinked network, which effectively restricts acetone diffusion into the cured coating. These findings demonstrate that PDTSO enhances the UV-curing process through its reactive functional groups, resulting in a higher degree of crosslinking and improved network compactness. The excellent agreement between the increased gel content and the reduced swelling ratio confirms the enhanced crosslinking efficiency of the PDTSO-modified coatings, which is expected to improve the solvent resistance and structural integrity of the cured films.^9,53^ These findings directly demonstrate that PDTSO does more than only increase the degree of entanglement; rather, it fosters the development of a more cohesive and interconnected network.

**Table 2:** A table compiling a number of analyses that highlight the mechanical performance of UV-cured polymer coatings

Samples (wt% PDTSO)	Gel fraction (%)	Swelling (%)	Contact angle (°)	Scratch resistance (kg)	Flexibility (5 mm)	Adhesion	Gloss (60°), GU	Impact (50 cm)	Impact (100 cm)
0	84.0 ± 0.8	28.0 ± 0.7	69.2° ± 3.8	0.85 ± 0.19	Failed	2B	70° ± 3	Pass	Failed
1	88.5 ± 1	22.0 ± 0.6	71.6° ± 2.9	1.25 ± 0.15	Pass	3B	78° ± 2.8	Pass	Pass
2	91.7 ± 1	16.0 ± 0.5	74.3° ± 2.1	1.5 ± 0.12	Pass	3B	93° ± 2.2	Pass	Pass
3	93.3 ± 0.5	10.0 ± 0.4	79.7° ± 1.5	2.0 ± 0.1	Pass	4B	96° ± 1.2	Pass	Pass
4	95.0 ± 0.4	7.0 ± 0.3	85.2° ± 1.2	2.4 ± 0.08	Pass	4B	99° ± 0.8	Pass	Pass

### 3.5. Contact angle measurement

Water contact angle (WCA) measurements were used to evaluate the wetting behavior of ESOA@TPGDA–PDTSO coatings. As the PDTSO content increased from 1 to 4 wt%, the WCA of the UV-curable epoxy acrylate coatings increased from 69° to 85° (Fig. 6B), indicating enhanced hydrophobicity. The rise in static contact angle with increased PDTSO content can be attributed to chemical and topographic effects caused by surface roughness, which improves the material's self-wetting capabilities. According to the findings, the large increase in contact angle suggests that PDTSO principally improves the coating's self-wetting capabilities by integrating long alkyl chains (of oleic acid) and siloxane segments, hence lowering surface energy. While surface roughness may contribute to the observed increase, chemical alteration of the surface structure is the primary cause. The long-chain fatty acids present in PDTSO contribute to this behavior by forming a barrier that limits water absorption and penetration into the polymer matrix. In addition, PDTSO promotes crosslinking during curing, resulting in a denser polymer network with reduced porosity, thereby restricting the diffusion of water and corrosive ions through the coating.^54^

**Fig. 6:**
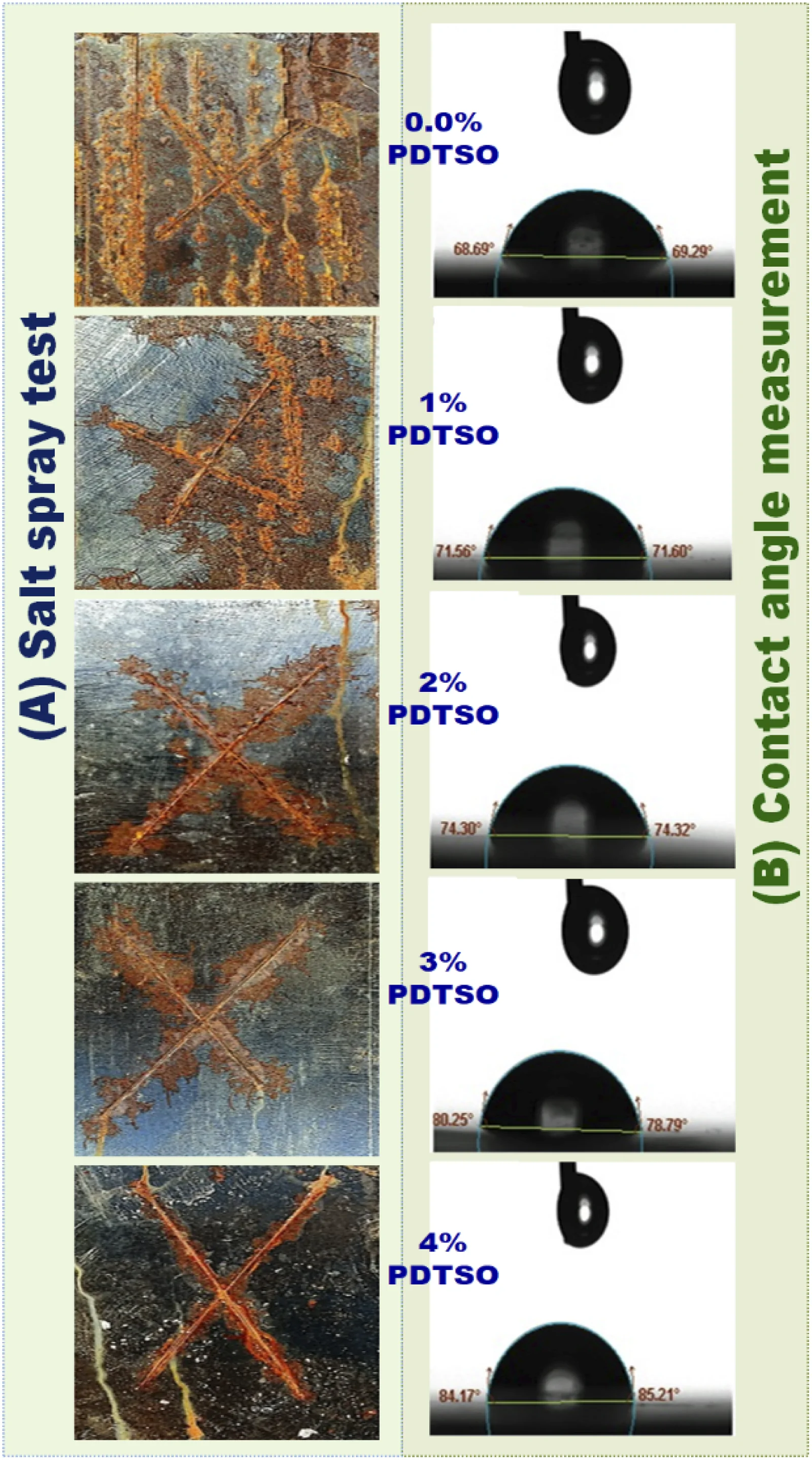
The UV-cured polymer coatings' surface features, including (A) the salt spray test; and (B) WCA measurements of the synthesized thin films of the UV-curable ESOA@TPGDA–PDTSO fabricated at different concentrations of the PDTSO (0, 1, 2, 3, and 4 wt%).

Surface roughness can also produce energy barriers that stabilize the contact line, resulting in enhanced contact angle hysteresis, which is regulated by PDTSO content. However, the results confirm that increasing PDTSO promotes a more uniform surface with reduced topographic inconsistencies. Scanning electron microscopy (SEM) analysis (Fig. 5) shows that PDTSO-containing coatings exhibit smoother and more uniform surfaces compared to the control coating. The reduced contact angle hysteresis suggests that PDTSO minimizes surface defects during curing, thereby enhancing barrier properties and providing more stable corrosion protection. However, the findings verify that raising PDTSO encourages a more homogeneous surface with fewer topographic irregularities. According to the SEM study (Fig. 5), PDTSO-containing coatings have smoother, more uniform surfaces than the control coating. The findings reflect that PDTSO minimizes surface defects during curing, which improves barrier properties and provides more reliable corrosion protection.

Overall, the structural enhancements resulting from PDTSO incorporation improve corrosion resistance by reducing moisture absorption and limiting electrochemical deterioration processes. Consequently, surfaces exhibit low-impact behavior, leading to more uniform wetting and reduced susceptibility to localized corrosion from defects. Improved thermal and chemical stability also contributes to the silicone amine's protective barrier capabilities. PDTSO-modified UV-curable ESOA@TPGDA coatings have enhanced water resistance and great durability due to the synergistic impact of increased hydrophobicity, which enhances barrier performance and makes them appropriate for industrial applications, particularly in humid and marine environments.

### 3.6. Salt spray test

As shown in Fig. 6A, after 500 h of salt spray exposure, the control coating (0 wt% PDTSO) exhibited severe corrosion, extensive rust propagation beyond the X-shaped scribe, and a 2G rating (ASTM D1654), indicating >15% corroded area. The addition of 1 wt% PDTSO significantly reduced corrosion, with rust mainly confined to the scribe lines and only minor delamination, resulting in a 4G rating. At 2–3 wt% PDTSO, rust coverage decreased to <3% with no observable delamination, yielding 5G and 6G ratings, respectively. The optimum formulation containing 4 wt% PDTSO achieved a 7G rating, showing only trace rust (<1%) within the scribe and confirming excellent barrier performance. This improvement is attributed to enhanced cross-linking density and molecular packing during UV curing, which reduce microstructural defects and limit water and chloride ion penetration.^44^

The presence of polar functional groups per unit volume must be taken into account, even though increasing cross-linking density greatly enhances the coating's physical insulating qualities. These groups have a dual competing effect: a higher concentration of polar groups improves the polymer matrix's affinity for water, while a denser network decreases free volume and limits water diffusion. The substantial drop in water absorption and the rise in the contact angle from 69° to 85° with increased PDTSO concentration show that the physical barrier effect predominates under the conditions under study, according to our findings. This implies that even with polar groups present, the surface wettability is decreased. Additionally, the hydrophobic nature of the long fatty acid chains in PDTSO may reduce the water attraction caused by the ester and ether groups. Furthermore, water penetration is limited by the hydrogen bond networks formed by the silicon–amine functional groups. Despite the presence of polar groups, this synergistic combination of improved cross-linking, hydrophobic fatty acid chains, and hydrogen bond interactions improves the barrier's overall effectiveness.

### 3.7. The physico-chemical and mechanical properties of cured coated films

To ascertain the suitability of the UV-cured epoxy acrylate coatings for protective applications, their mechanical and surface properties, including scratch resistance, flexibility, adhesion, gloss, and impact resistance, were evaluated.

As shown in Table 2 and Fig. 7, the scratch resistance increased steadily from 0.85 kg for the control coating to 2.4 kg for the formulation containing 4 wt% PDTSO. This improvement is attributed to the fatty acid chains of PDTSO, which promote higher cross-link density within the cured coating.^55^ Gel content results further confirmed enhanced network integrity and resistance to mechanical wear.

**Fig. 7:**
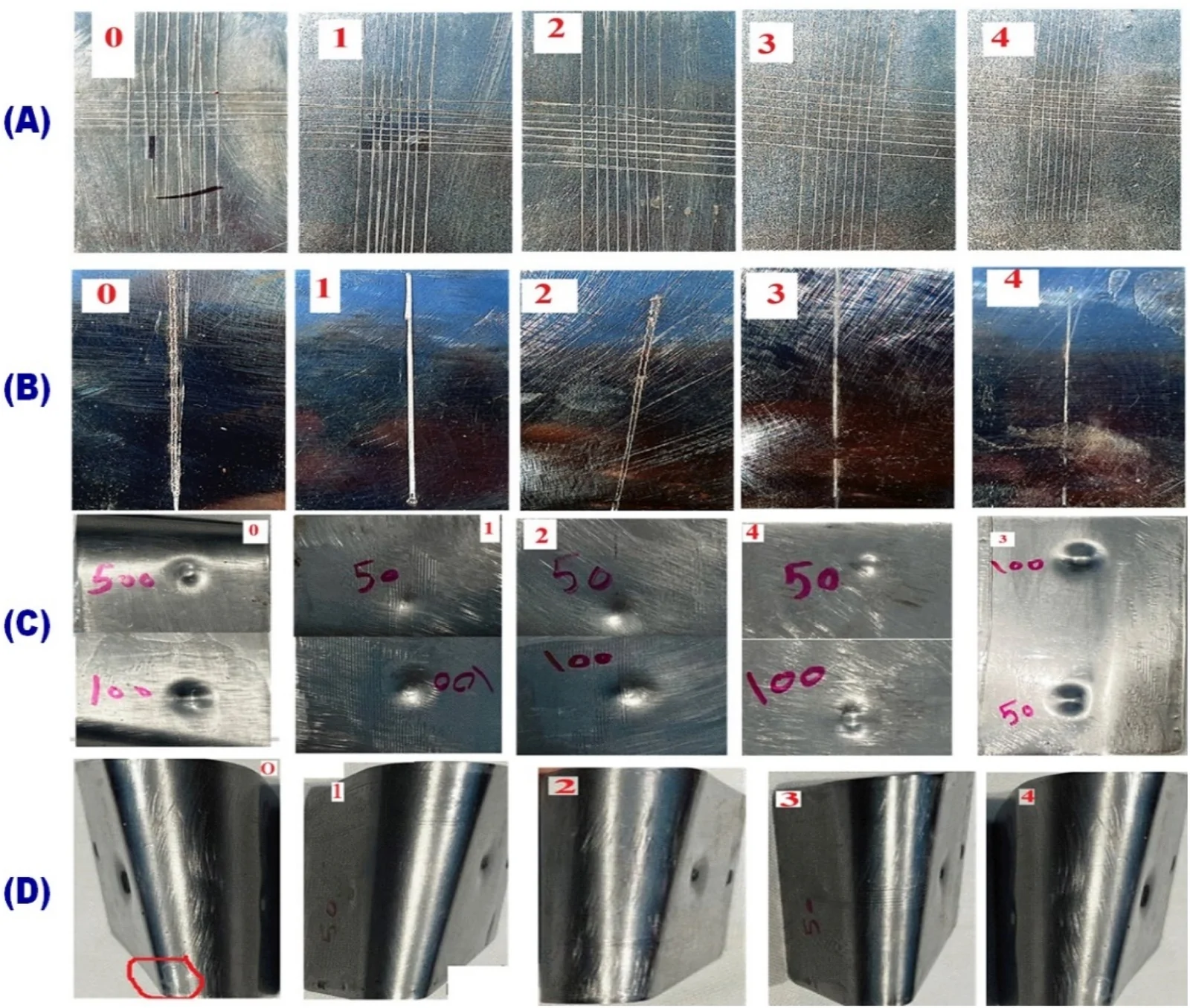
The mechanical and surface properties of the UV-cured coatings including (A) adhesion strength, (B) scratch resistance, (C) impact resistance, and (D) flexibility.

Flexibility was assessed using a 5 mm cylindrical mandrel. The control coating failed due to cracking and adhesion loss, indicating insufficient cross-linking and incomplete polymer conversion. This was probably due to insufficient cross-linking and incomplete polymerization, resulting in a brittle, hard coating. In contrast, all PDTSO-containing coatings passed the test without visible damage. The long hydrocarbon chains of PDTSO act as plasticizing segments, increasing elasticity and reducing brittleness, thereby improving coating flexibility. Overall, PDTSO provides coatings with enhanced scratch resistance and flexibility, ensuring durability under harsh conditions.

Adhesion to metal substrates was evaluated using the standardized cross-cut adhesion test. All coatings containing PDTSO exhibited excellent adhesion, whereas the control coating showed a lower rating of 2B, indicating partial detachment or flaking. The improved adhesion is attributed to the polar functional groups of PDTSO and siloxane (–Si–O–) linkages, which promote strong interfacial interactions with the metal surface.^56^

Gloss measurements at a 60° angle ranged from 70 to 99 gloss units, reflecting the surface appearance of the cured coatings.^57^ As shown in Table 2, the epoxy acrylate coating without PDTSO exhibited the lowest gloss value. Acting as a reactive diluent, PDTSO improved surface leveling and reduced formulation viscosity during curing,^58^ resulting in smoother, more homogeneous surfaces and enhanced gloss. In addition, PDTSO-induced cross-linking contributed to improved surface uniformity and gloss performance. To replicate mechanical shock, the coatings' impact resistance was evaluated by dropping a 1 kg load from heights of 50 and 100 cm. PDTSO-modified coatings exhibited excellent impact resistance at both heights due to their increased cross-link density and the flexibility imparted by the long aliphatic chains of PDTSO. These features improved energy absorption during impact. In contrast, the control coating (epoxy acrylate coating without PDTSO) passed only the 50 cm test and failed at 100 cm because of poor network development and weaker substrate adhesion. These results demonstrate the effectiveness of PDTSO in enhancing the gloss, adhesion, and mechanical durability of UV-curable coatings.^10,59^

### 3.8. Impact of tertiary amine on curing rate

The influence of tertiary amine on the curing rate of UV-cured coatings is significant, particularly when used with a Norrish type II photoinitiator. Norrish type II photoinitiators require a hydrogen donor, commonly referred to as a co-initiator, to generate free radicals through hydrogen abstraction, thereby initiating the polymerization of oligomers and monomers *via* the Norrish type II mechanism. Tertiary amines are highly efficient hydrogen donors and are therefore widely used in combination with Norrish type II photoinitiators.^60^

In our study, a fatty acid combined with tertiary amine was used as a reactive diluent in UV-cured coatings. The samples were exposed to UV light at a distance of 15 cm for 20 s at room temperature under air (without vacuum). The results showed that coatings containing PDTSO exhibited a faster curing rate and higher conversion than those without PDTSO. When exposed to UV light, benzophenone is excited to its triplet form and extracts an α-hydrogen atom from the tertiary amine, resulting in the production of an α-amino alkyl radical and a ketyl radical. The acrylate double bond polymerization is effectively initiated by the extremely reactive α-amino alkyl radical, while the ketyl radical primarily experiences termination reactions.

The presence of oxygen in the system strongly inhibits the elimination of free radicals. Oxygen suppresses free radical photopolymerization by interacting with catalyzed radicals to produce comparatively inactive peroxy-based radicals, which reduces double bond conversion.^61^ Formulations with tertiary amines maintain a greater conversion rate, whereas coatings without tertiary amines see a sharp decline in conversion rate. The α-aminoalkyl radicals generated from tertiary amines that preferentially react with dissolved oxygen, consuming it through the production of peroxy-based radicals and shortening the duration of oxygen inhibition. These peroxy-based radicals do not directly promote chain growth, but their oxygen-absorbing action lowers the amount of dissolved oxygen available to quench the scattered radicals, facilitating more effective photopolymerization. Consequently, the presence of tertiary amines enhances monomer conversion and facilitates the formation of denser crosslinked polymer network (Fig. 8).^61^

**Fig. 8:**
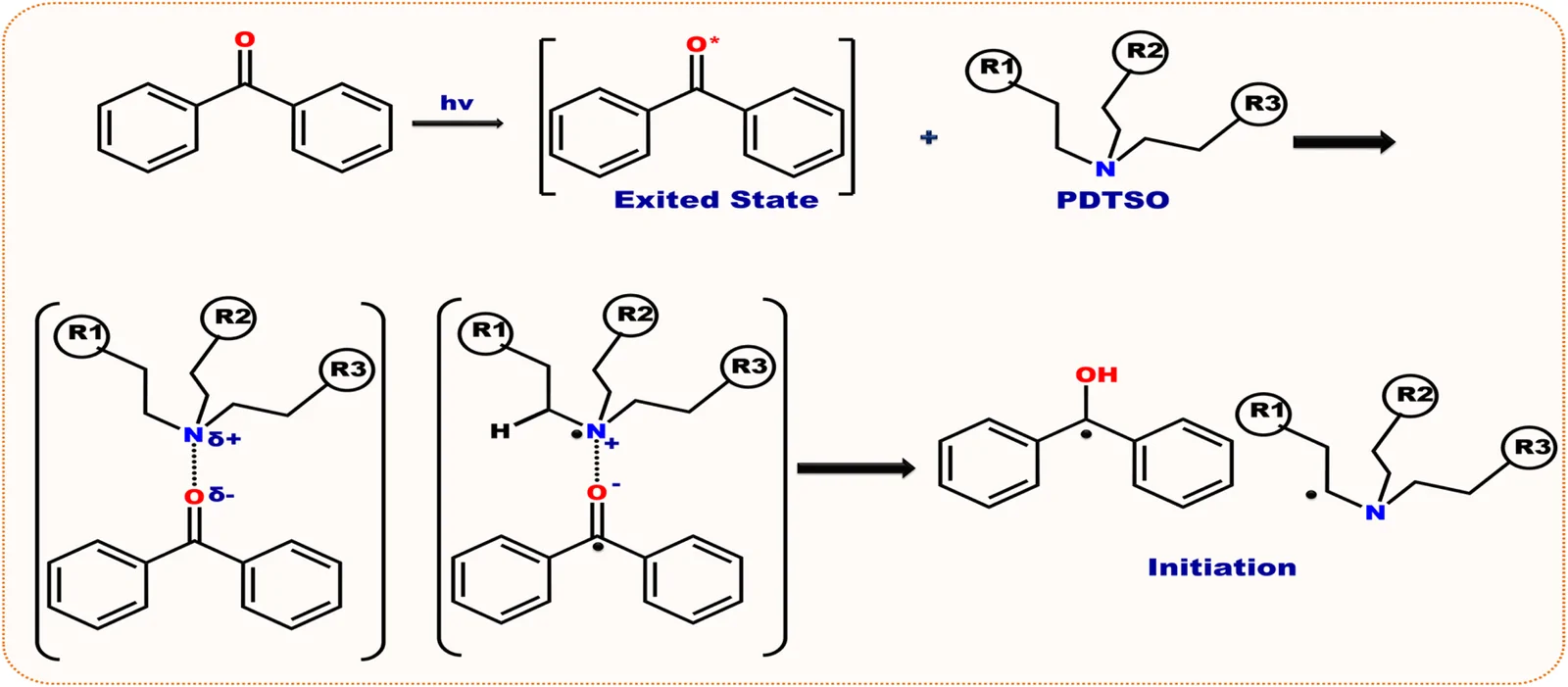
Mechanism of benzophenone in UV curing and the function of PDTSO as a hydrogen abstractor.

### 3.9. Electrochemical measurements

#### 3.9.1. Open-circuit potential (OCP) measurements

An important source of information on the effect of different PDTSO concentrations on the anticorrosive behavior of UV-cured epoxy acrylate coatings on functionalized metallic surfaces in 3.5 wt% NaCl solution is provided by the open-circuit potential (OCP) curves (Fig. 9).

**Fig. 9:**
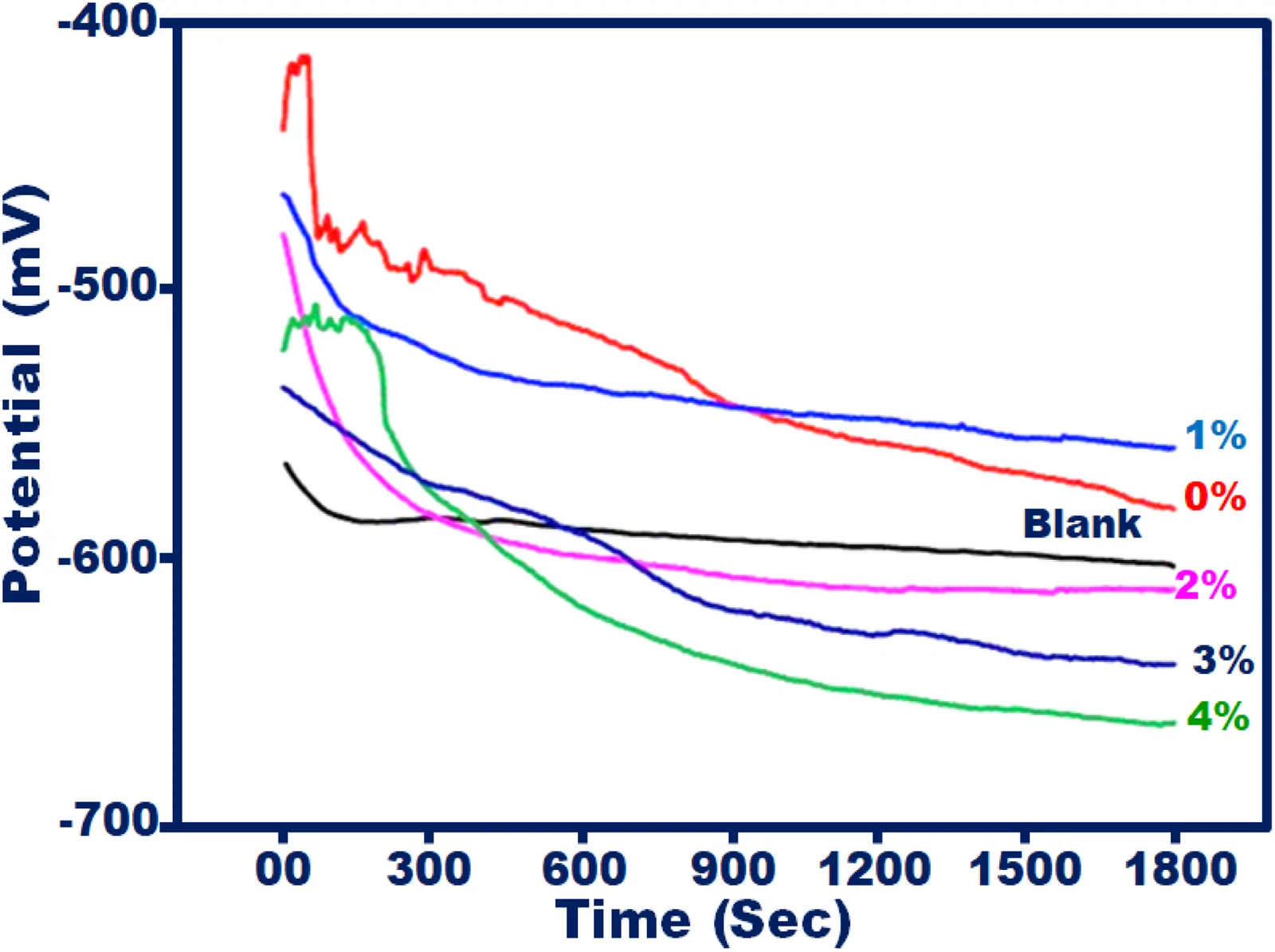
OCP–time curves for UV-cured coatings without and with different concentrations of poly(di-triethanolaminesiloxane-oleate) (PDTSO).

The uncoated sample showed a negative OCP (−580 mV *vs.* SCE), which is typical of active metal dissolution in chloride environments. Utilizing of the control ESOA@TPGDA coating (0% PDTSO), the OCP shifted to approximately −550 mV, indicating the formation of a polymer barrier that functions as an anodic-type inhibitor, suppresses anodic dissolution, and partially isolates the steel surface from the electrolyte. Instead of showing a monotonic positive shift, the OCP values varied with concentration after PDTSO was added. A slight additional positive shift to about −540 mV was caused by the presence of 1 weight percent PDTSO. It's interesting to note that raising the PDTSO concentration from 2 to 4 weight percent caused a progressive cathodic shift in OCP with greater negative steady-state potential values, reaching about −660 mV at 4 wt%. These changes indicate that the addition of PDTSO modifies the electrochemical equilibrium at the coating/metal interface. Importantly, the cathodic OCP shift at higher PDTSO concentrations does not indicate reduced corrosion protection, but considered for describing the interfacial electrochemical behavior.

The improvement of corrosion protection provided by PDTSO is attributed to the formation of a denser and less permeable polymer network due to PDTSO's hydrophobic and crosslinking properties. Where, the cathodic shift associated with the increased hydrophobicity and crosslinking density of the polymer network, which limits oxygen diffusion and suppresses the cathodic oxygen reduction reaction. The increased corrosion resistance is also related to the presence of heteroatoms (N, O, and Si) in the polymer matrix, which can coordinate with vacant iron orbitals on the metal surface, enhancing adhesion through stable interfacial bonding. Nitrogen and oxygen act as electron donors, forming coordination complexes that stabilize the coating and reduce delamination or degradation. The combined effect of improved adhesion and hydrophobicity enhances the barrier properties, leading to reduced electrochemical activity and the formation of a more durable protective layer under harsh environmental conditions.

#### 3.9.2. Electrochemical impedance spectroscopy (EIS)

A suitable and non-destructive technique for understanding the electrochemical processes occurring at the interfaces of the coating and the electrolyte and between the metal and the coating is electrochemical impedance measurement (EIS). This offers quantifiable data on polymer coatings' degradation processes and protective efficacy. To evaluate the protective performance of PDTSO and understanding the corrosion inhibition mechanism of coatings manufactured with different concentrations of PDTSO, the data were presented in three different formats: Nyquist plots and Bode plots, which include representations of both magnitude (log|*Z*| *vs.* log *F*) and phase angle (−*θ vs.* log *F*), as shown in Fig. 10. Table 3 provides a summary of the relevant results.

**Fig. 10:**
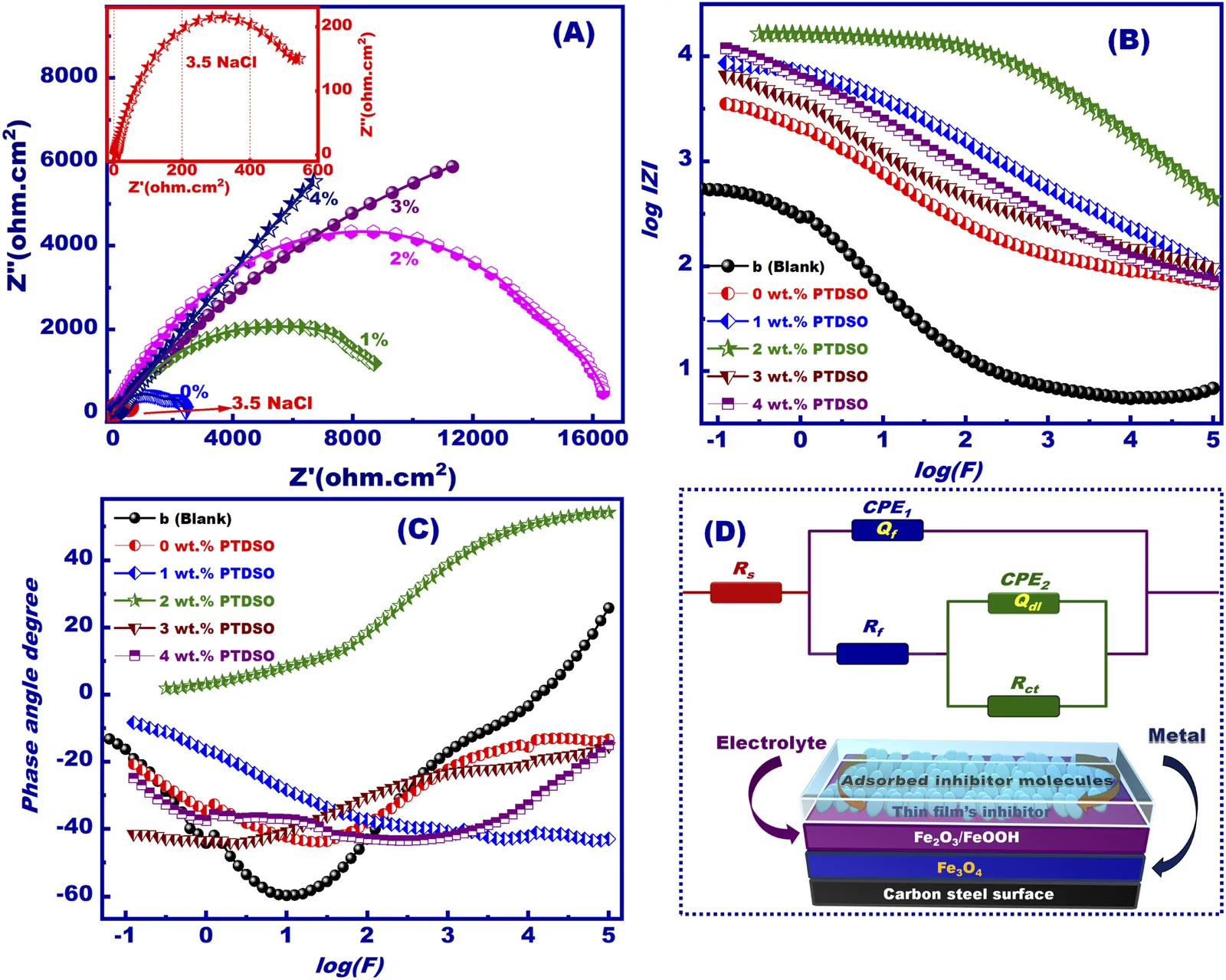
EIS analysis of uncoated and coated mild steel in 3.5 wt% NaCl solution at 30 °C: (A) Nyquist plots for ESOA@TPGDA coatings with 0–4 wt% PDTSO; (B) Bode impedance magnitude plots (log|*Z*| *vs.* log *F*); (C) Bode phase angle plots (−*θ vs.* log *F*) showing two time constants for coated samples; and (D) equivalent circuit used for fitting data for impedance.

**Table 3:** EIS parameters for the UV-cured coatings at various concentrations of PDTSO

Sample (wt% PTDSO)	*R*_1_ (Ω cm^2^)	*Q*_1_ (S s^*n*^ cm^−2^)	*n*_1_	*R*_f_ (Ω cm^2^)	*C*_1_ (mF cm^−2^)	*Q*_2_ (S s^*n*^ cm^−2^)	*n*_2_	*R*_ct_ (Ω cm^2^)	*C*_dl_ mF^2^ cm^−1^	*C*_2_ (mF cm^−2^)	Slope	Phase angle
B	6	6.07 × 10^−4^	0.78	606	460 × 10^−3^	3.3 × 10^−2^	1.00	83	33.11	33.11	−0.177	−59.8
0	40	2.65 × 10^−6^	0.57	7126	8 × 10^−3^	2.6 × 10^−5^	0.81	6016	0.01683	0.017	−0.249	−43.82
1	91	1.32 × 10^−4^	0.42	574	4 × 10^−3^	1.9 × 10^−5^	0.82	6185	0.01187	0.012	0.122	−41.35
2	74	1.19 × 10^−4^	0.32	130	2 × 10^−5^	1.6 × 10^−5^	0.80	7900	0.00954	0.009	0.042	−53.9
3	78	1.47 × 10^−8^	0.97	81	1 × 10^−5^	1.3 × 10^−5^	0.75	10 030	0.006592	0.007	0.305	−44.01
4	28	5.83 × 10^−5^	0.41	76	2 × 10^−5^	1.1 × 10^−6^	0.70	21 006	0.000219	0.006	−0.346	40.45

Nyquist plots for uncoated mild steel and UV-cured ESOA@TPGDA coatings with varying PDTSO concentrations (0–4 wt%) immersion in a 3.5 wt% NaCl solution at 30 °C are displayed in Fig. 10A. The uncoated mild steel electrode shows the smallest semicircle in the Nyquist plot in the high-frequency region, indicating low charge transfer resistance (*R*_ct_ = 83 Ω cm^2^) and poor corrosion resistance (high corrosion susceptibility to electrochemical attack) due to full exposure of the metallic surface to the corrosive medium in the absence of a protective layer.

The control ESOA@TPGDA coating (0% PDTSO) shows a slight increase in semicircle diameter (*R*_ct_ = 6185 Ω cm^2^), indicating limited corrosion protection due to formation of a basic physical barrier. However, its low crosslink density and permeability to ions and moisture still result in weak resistance. With increasing PDTSO content from 1 to 4 wt%, a progressive increase in semicircle diameter was observed, indicating enhanced impedance and improved charge transfer resistance, reaching values of 7900 Ω cm^2^ (2 wt%), 10 030 Ω cm^2^ (3% wt%), and 21 006 Ω cm^2^ (4 wt%). This notable rise in charge transfer resistance associated with the increase in the PDTSO content is in line with OCP findings and validates the improved barrier features and corrosion protection performance of PDTSO-modified coatings. This improvement is attributed to the formation of a dense, highly crosslinked protective coating that impedes ion transport and reduces corrosion current density.

Analysis of the Nyquist diagram of 3% and 4% PDTSO coatings reveals a Warburg-type linear phase in the low-frequency region, indicating the transport of corrosive species, such as H_2_O, O, and Cl ions, through the dense polymer matrix *via* diffusion control. Therefore, mass transfer takes the place of charge-transfer control as the rate-limiting step. This behavior demonstrates that high PDTSO concentrations produce extremely dense and impermeable coatings that successfully prevent ion penetration. The crosslinking density and molecular weight of the coating increase with increasing PDTSO content in the polymer matrix. These structural improvements form denser polymer networks with enhanced barrier properties, effectively hindering chloride ion penetration. This is reflected in the impedance spectra by an increase in resistive components with rising PDTSO concentration. The increased crosslinking density and decreased free volume are associated with enhanced barrier efficiency, which is supported by X-ray diffraction data showing a decrease in interlevel distance with increasing PDTSO content.

The Bode magnitude plots (log|*Z*| *vs.* log *F*) in Fig. 10B clearly show the inhibitory action of PDTSO against corrosion. The impedance magnitude (|*Z*|) of PDTSO-containing systems is significantly greater than that of the uncoated mild steel reference solution in the low-frequency region, which is linked to the interfacial polarization resistance. Additionally, |*Z*| gradually rises with PDTSO concentration, demonstrating the substantial concentration dependence of the protective effectiveness. Increased polarization resistance and, thus, increased corrosion resistance are indicated by the comparatively steady impedance response at low frequencies.

Fig. 10C demonstrated that the phase-angle graphs (−*θ vs.* log *F*) shed more light on the surface heterogeneity and interfacial electrochemical processes. The uncoated mild steel system shows a wide phase-angle maximum, which is indicative of a corrosion process that is primarily controlled by charge transfer. The phase-angle maximum broadens and changes toward higher values when PDTSO is added, especially at increasing concentrations. This change indicates the creation of a compact protective layer at the metal/electrolyte interface and represents improved capacitive behavior. Increased surface heterogeneity brought on by PDTSO inhibitor adsorption and the potential coexistence of charge-transfer and film-related time constants could be the cause of peak broadening. However, the lack of distinct peaks indicates a significant coupling between these processes. Overall, PDTSO adsorption and the creation of a barrier that prevents active corrosion sites are confirmed by the increased impedance, charge-transfer resistance, and phase-angle changes. Surface-film development is the primary inhibitory mechanism, as further evidenced by the concentration-dependent response.

Based on the Bode phase angle analysis, for accurate interpretation of EIS information, choosing an appropriate equivalent electrical circuit (EEC) is essential due to the capability to distinguish the individual influences of diffusion processes, charge transfer reactions at the interface, and resistance of the coating. The *R*(*Q*(*R*(*Q*(*R*)))) circuit was selected for UV-cured polymer coatings deposited on mild steel and tested in 3.5% NaCl solution based on theoretical considerations and statistical goodness-of-fit parameters (Fig. 10D). This study offers a trustworthy framework for evaluating the protective effectiveness of UV-cured PDTSO-modified coatings and connecting circuit parameters to important characteristics including adhesion strength, hydrophobicity, and cross-linking density. Key parameters such as *R*_ct_, *R*_f_ (coating resistance), *C*_c_ (coating capacitance), and the constant phase element of the double layer (dl) provide insight into coating performance, where higher *R*_ct_ and *R*_f_ and lower *C*_dl_ (double-layer capacitance (*C*_dl_)) indicate better corrosion resistance. By taking into account the effects of coating porosity, non-ideal capacitive properties, electrochemical activity at the metal/coating interface, and diffusion-controlled transport through the porous polymer network, this four-time-constant model effectively explains the heterogeneous behavior of the coated system. The EIS results verify that *R*(*Q*(*R*(*Q*(*R*)))) accurately characterizes the electrochemical behavior and protective performance of the designed environmentally friendly coatings when combined with electrodynamic polarization and salt-spray results.

Furthermore, a corresponding decrease in *C*_dl_ is observed with increasing PDTSO content, suggesting a denser and more hydrophobic polymer matrix with reduced dielectric activity at the interface. These improvements are mainly attributed to the synergistic effect of Si, O, and N functional groups in PDTSO, which enhance adhesion, increase crosslink density, and limit diffusion of corrosive species, thereby improving electrochemical stability and durability.^13,62^

A slight decrease in the *n*-value (Table 3) is observed and is typically associated with surface roughness and microstructural imperfections of the carbon steel substrate. However, this effect is mitigated by the uniform deposition of the polymer film, which reduces surface heterogeneity and produces a smoother, more cohesive barrier layer. The decrease in the admittance parameter (*Y*_o_) with increasing PDTSO content is attributed to the replacement of water and corrosive ions by polymer chains at the interface, reducing ion mobility and electrochemical activity and further enhancing corrosion protection. Overall, these results confirm that PDTSO significantly improves the structural and electrochemical performance of the coating.

The *R*_ct_, and *C*_dl_ are connected in parallel in the equivalent circuit model. Analyzing *C*_dl_ values requires the use of the constant phase element (CPE) parameters, particularly *Y*_o_ and *n*, which stand for the magnitude and an adjustable parameter, respectively.^63^ To calculate the *C*_dl_, the following equation was applied:^13,64^*C*_dl_ = (*Y*_o_*R*_ct_^(1−*n*)^)^1/*n*^

This equation further validates the improved corrosion protection offered by the PDTSO-modified coatings by considering the coating system's less-than-ideal capacitance behavior.

#### 3.9.3. The potentiodynamic polarization (PDP) measurements

The electrodynamic polarization curves of uncoated and coated mild carbon steel samples in a 3.5 wt% sodium chloride solution are shown in Fig. 11, with the corresponding electrochemical parameters summarized in Table 4. The corrected *E*_corr_ results are now completely consistent with the OCP patterns noted in Section 3.9.1 after a thorough re-analysis of the polarization data using consistent Tafel extrapolation parameters.

**Fig. 11:**
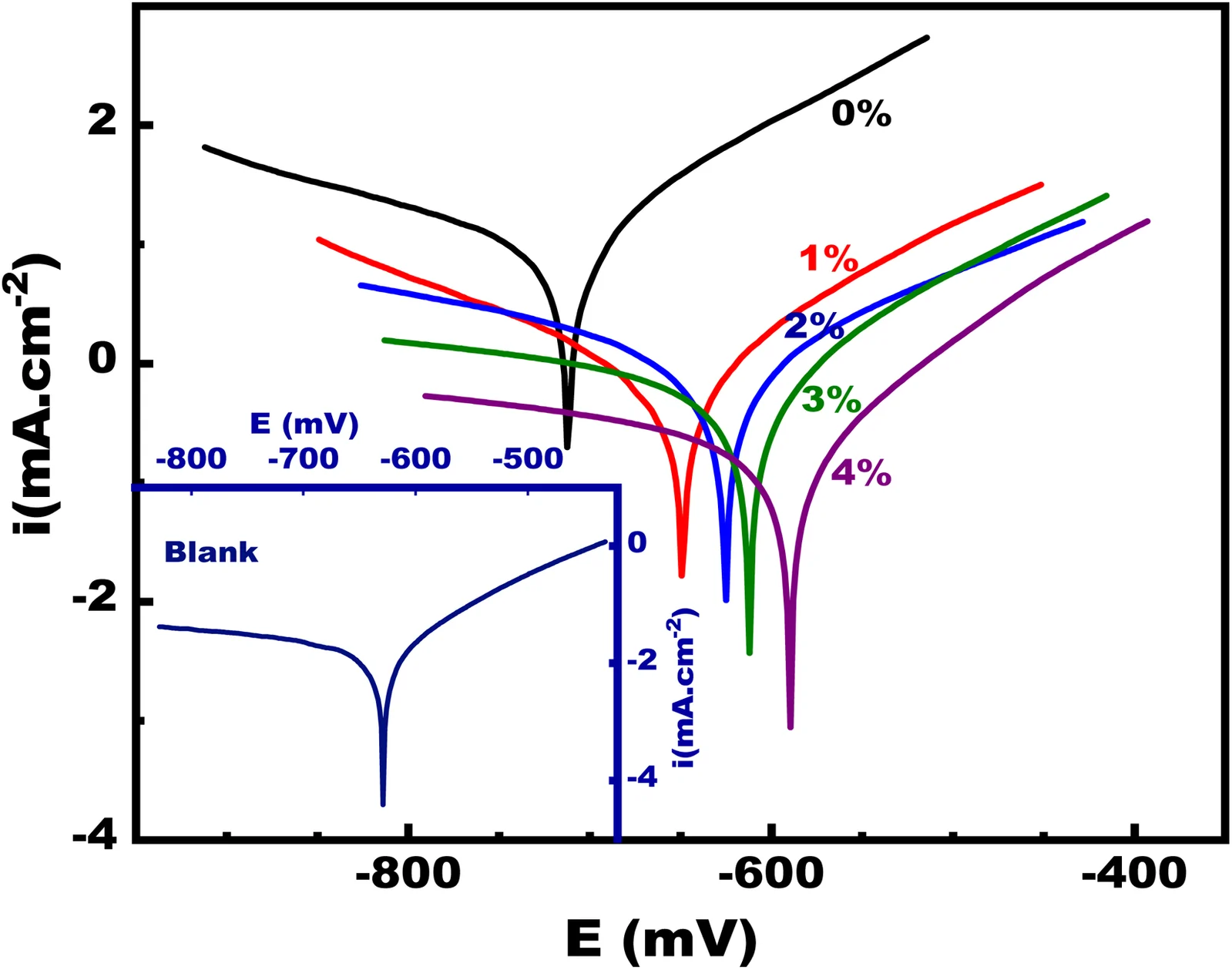
Potentiodynamic polarization curves for the UV-curable polymer coatings included varying PDTSO concentrations.

**Table 4:** Tafel polarization parameters for the UV-cured coatings at various concentrations of PDTSO

Sample (wt% PDTSO)	*E*_corr_ (mV)	*i*_corr_ (µA cm^−2^)	*R*_p_ (kΩ cm^2^)	*β*_a_ (mV dec^−1^)	*β*_c_ (mV dec^−1^)	Corrosion rate (µm·per year)	*θ*	*η* (%)
Blank	−629.0	18.70	1.81	103.5	−544.2	218.62	—	—
0	−711.9	5.48	2.71	97.5	−186.6	64.13	0.71	71
1	−649.1	1.58	37.98	151.8	−256.5	18.46	0.914	75
2	−625.4	0.948	36.66	159.3	−267.4	11.09	0.951	82
3	−612.2	0.637	56.00	159.2	−487.6	7.45	0.966	85
4	−633.7	0.177	172.95	166.4	−217.4	2.07	0.990	90

When the control ESOA@TPGDA coating (0% PDTSO) was utilized, *i*_corr_ decreased and *E*_corr_ was moved to a negative value, showing anodic-type inhibition through physical barrier development. The polarization curves show a progressive decrease in the corrosion current density (*i*_corr_) with increasing PDTSO concentration, demonstrating a notable increase in corrosion resistance. The *i*_corr_ dropped from 18.70 µA cm^−2^ for the uncoated steel to 0.177 µA cm^−2^ for the coating with 4 wt% PDTSO, corresponding to an inhibition efficiency of 90% as shown in Table 4.^65,66^ This increase in inhibitory efficacy is linked to anodic protection thru PDTSO functional group chemisorption. These findings demonstrate that PDTSO-modified UV-cured epoxy acrylate coatings greatly improve electrochemical stability and corrosion inhibition in saline environments, highlighting PDTSO as an effective additive for improving both electrochemical and barrier protection in polymer coatings.

The anodic and cathode branches of the coated samples generally remain parallel to those of the uncoated sample, indicating that the underlying corrosion mechanism is not significantly altered by the addition of PDTSO. On the contrary, both the anodic metal dissolution and cathodic reduction reactions are effectively suppressed, as evidenced by the simultaneous decrease in current density. Additionally, the relatively slight variations in *E*_corr_ and minimal change in Tafel slopes indicate that PDTSO acts as a mixed-type corrosion inhibitor, providing protection primarily through the formation of an effective physical barrier layer and reduced charge transfer, rather than changes in electrochemical reaction pathways. The dense, crosslinked polymer network restricts the penetration of water and chloride ions. Moreover, Si-, O-, and N-containing functional groups improve coating adhesion and interfacial stability through interactions with the steel surface, thereby reducing charge transfer and providing durable long-term corrosion protection.

The inhibition efficiency (*η*%) was calculated from the corrosion current densities using following equation:
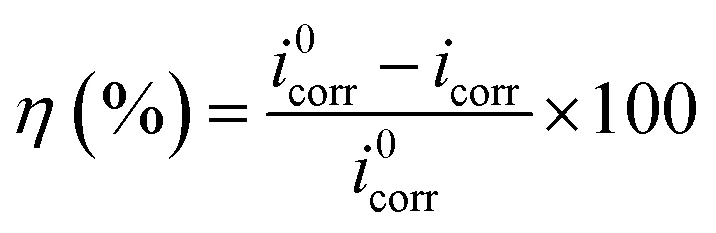
where *i*^0^_corr_ and *i*_corr_ are the corrosion current densities of the uncoated and coated samples, respectively.

### 3.10. Corrosion mechanism

PDTSO-modified UV-cured epoxy acrylate coatings provide improved corrosion protection through a synergistic combination of chemical processes, electrochemical inhibitory mechanisms, and physical barrier effects. Diverse mechanisms for corrosion inhibition have been suggested based on thorough investigation. Multifaceted corrosion inhibition mechanisms have been proposed based on thorough investigation. The development of a physical barrier, improved chemical adsorption and adhesion at the interface, concentration-dependent electrochemical inhibition (anodic at low PDTSO concentrations and cathodic at high PDTSO concentrations), water exclusion due to hydrophobicity, and network thickening from cross-linking are some of the interrelated factors that these mechanisms rely on to provide superior corrosion protection.

By creating a dense, highly crosslinked polymer network that keeps mild steel away from the corrosive electrolyte, the ESOA@TPGDA–PDTSO coating effectively prevents corrosion. While siloxane segments improve hydrophobicity and coating compactness, reducing the penetration of water and chloride ions, PDTSO's tri-amine groups encourage cross-linking. Functional groups comprising Si, O, and N enhance interfacial adhesion and suppress charge transfer at the coating/steel interface. The increase in gel content from 84.0% for the unmodified coating to 95.0% at 4% PDTSO supports the improved cross-linking, suggesting the creation of a tightly interconnected 3D network with decreased free volume and molecular mobility. XRD data demonstrate structural densification, with interchain distance decreasing from 4.68 to 4.40 Å as PDTSO increased from 0 to 4%. This compact molecular arrangement restricts diffusion pathways for water, dissolved oxygen, and chloride ions, thereby reducing electrolyte penetration and electrochemical activity at the coating–metal interface.

As electron donors, the heterogeneous N, O, and Si atoms in PDTSO encourage chemisorption and the creation of stable coordination bonds between the electron pairs of these atoms and the iron's 3d orbitals, resulting in a stable interlayer with strong adhesion. The enhanced adhesion was verified by cross-cutting tests, where PDTSO (3 and 4 wt%) layers achieving a 4B rating as compared to the control layer's 2B rating. This improved adhesion minimizes layer separation, bubble formation, and substrate erosion in chloride-rich conditions. EDS examination also validated the homogeneous distribution of silicon and nitrogen, showing that PDTSO was dispersed uniformly and had excellent interlayer bonding.

OCP and PDP analysis support the concentration-dependent behavior of PDTSO's electrochemical inhibitory mechanism. PDTSO primarily functions as an anodic inhibitor at low concentrations (0–1 weight percent) by preventing iron dissolution *via* anodic-site passivation and protective layer generation. By blocking active dissolving sites, lowering the anodic exchange current density, and supplying electron density to the iron surface, chemisorbed PDTSO molecules stabilize Fe^2+^/Fe^3+^ species. Changes in OCP and successful suppression of the oxygen reduction reaction indicate a shift in the inhibitory mechanism toward cathodic control at higher concentrations (2–4 weight percent). The thick, crosslinked polymer network created by PDTSO is linked to this characteristic. While siloxane groups aid in surface passivation and stability, its hydrophobic nature limits electrolyte penetration and electron transport at the coating–metal interface. Therefore, as PDTSO concentration increases, the shift from anodic to cathodic inhibition reflects changes in dominating electrochemical contacts, molecular organization, and surface coverage.

Generally, The OCP measurements indicate changes in the electrochemical equilibrium at the coating/electrolyte interface, while *E*_corr_ values obtained from polarization provide a more direct kinetic measure of corrosion activity. The EIS results, in turn, demonstrate improved barrier properties through increased coating and charge-transfer resistance. Furthermore, PDTSO functions as a mixed-type corrosion inhibitor by suppressing both anodic and cathodic processes, as demonstrated by the notable decrease in corrosion current density seen in the polarization tests. As a result, the coating with 4 wt% PDTSO demonstrated the best corrosion protection, with a 90% inhibition efficiency.

### 3.11. Evaluation in light of previous studies

Fayyad *et al.*^67^ investigated corrosion-resistant coatings based on oleic acid-grafted chitosan in NaCl solution under the same immersion conditions used in this study. The addition of graphene oxide nanoparticles reduced the *i*_corr_ value from 7.7 to 4.4 µA cm^−2^. Although corrosion protection improved, our UV-curable coating exhibited a much lower *i*_corr_ value (0.17 µA cm^−2^), demonstrating superior corrosion resistance without requiring nanoparticles.

Similarly, Deyab *et al.*^68^ reported comparatively lower protective performance for epoxy resin systems without corrosion-inhibiting pigments or nanoparticles. In contrast, our nanoparticle-free coating provides excellent corrosion protection while offering rapid UV curing and a solvent-free, eco-friendly composition. Unlike conventional alkyd resin coatings, which require fatty acid modification and long curing times under solvent-rich conditions, the present UV-curable epoxy acrylate system provides a more sustainable and time-efficient alternative. By applying green chemistry principles, it combines high performance with environmentally friendly and energy-efficient coating technology.

## 4. Conclusion

UV-curable coatings are known for their superior corrosion resistance, affordability, and environmental sustainability. The efficiency of their application is constrained by the ongoing need for better mechanical qualities and thermal stability. In this work, different percentages of PDTSO were added to UV-curable coatings based on ESOA@TPGDA in order to create a dense, densely crosslinked polymer matrix that would increase the corrosion resistance of coatings. The cured films underwent a thorough examination that included testing for mechanical, chemical, physical, and corrosion resistance. According to the results, the addition of PDTSO enhanced crosslinking density and molecular flexibility within the polymer network, facilitating effective polymer stacking during UV curing. Due to its functional groups, which allow for more widespread polymer crosslinking and result in coatings that are more durable and chemically resistant, this pattern showed that PDTSO actively catalyzes the curing procedure.

## Conflicts of interest

There are no conflicts to declare.

## Data Availability

All data generated and analyzed during this study are included in this published article. Characterization data (including FTIR, ^1^H NMR, SEM, XRD patterns, salt spray test; and WCA measurements) are provided within the main manuscript. Raw experimental data, including the original spectroscopic and electrochemical files, are available from the corresponding authors upon reasonable request. The authors confirm that no additional unpublished data or code were used in this study.
